# Targeting late-stage non-small cell lung cancer with a combination of DNT cellular therapy and PD-1 checkpoint blockade

**DOI:** 10.1186/s13046-019-1126-y

**Published:** 2019-03-11

**Authors:** Linan Fang, Dalam Ly, Si-si Wang, Jong Bok Lee, Hyeonjeong Kang, Hao Xu, Junlin Yao, Ming-sound Tsao, Wei Liu, Li Zhang

**Affiliations:** 1 Department of Thoracic Surgery, The First Hospital of Jilin University, Changchun, China; 2 Toronto General Hospital Research Institute, University Health Network, Toronto, ON Canada; 3 Department of Immunology, University of Toronto, Toronto, ON Canada; 4 Department of Translational Medicine, The First Hospital of Jilin University, Changchun, China; 5 Princess Margaret Cancer Centre, University Health Network, Toronto, Ontario Canada; 6 Department of Laboratory Medicine and Pathobiology, University of Toronto, Toronto, ON Canada; 7 Present address: Sir Run Run Shaw Hospital, College of Medicine, Zhejiang University, Hangzhou, China; 8 Toronto General Research Institute, Princess Margaret Cancer Research Tower, 101 College St. Rm 2-807, Toronto, Ontario M5G 1L7 Canada

**Keywords:** Double negative T cells, Adoptive cell therapy, Lung cancer, NSCLC, Checkpoint blockade, Anti-PD-1, Nivolumab, Combination therapy

## Abstract

**Background:**

Though immune checkpoint blockade (ICB) against PD-1 has shown success in the treatment of lung cancer, not all patients respond. We have previously shown that adoptive transfer of double negative T (DNT) cells expanded from healthy donors can target leukemia but their role in treating established lung cancer is not clear. Here we explore the role of human DNT cells in targeting late-stage established lung cancer either alone or in combination with Nivolumab (anti-PD-1 antibody) and describe underlying mechanisms.

**Methods:**

DNT cells from resected lung cancer tissue of patients were analyzed by flow cytometry to determine their infiltration and PD-1 expression. Expansion capacity and anti-tumor function of lung cancer patient and healthy donor DNT cells were compared. Late-stage lung cancer xenograft models were developed to determine the anti-tumor effect of DNT cells alone or in combination with anti-PD-1 antibody, and the level of tumor-infiltrating DNT cells was quantified by histology and characterized by flow cytometry.

**Results:**

Patient-derived tumor infiltrating lymphocytes contained a lower frequency of DNT cells with a higher expression of PD-1 relative to normal lung tissue. Ex vivo expanded patient- and healthy donor-derived DNT cells showed similar levels of cytotoxicity against lung cancer cells in vitro. Healthy donor-derived DNT cells significantly inhibited the growth of late-stage lung cancer xenografts, which was further augmented by anti-PD-1 through increased DNT cell tumor infiltration.

**Conclusion:**

This study supports the use of DNT cells for adoptive cellular therapy against lung cancer either alone or in combination with anti-PD-1.

**Electronic supplementary material:**

The online version of this article (10.1186/s13046-019-1126-y) contains supplementary material, which is available to authorized users.

## Background

Lung cancer is the leading cause of cancer mortality worldwide, with 85% of patients diagnosed with non-small-cell lung cancer (NSCLC), many presenting at an advanced stage of the disease [1, 2]. For decades, platinum-based chemotherapy was the only available systemic therapy for advanced NSCLC. However, the median survival of chemotherapy-treated patients was a modest 8–10 months [3]. Immunotherapies, which harness the host immune response to treat cancers have recently generated great excitement in lung cancer treatment, but currently approved therapies, such as immune checkpoint blockades (ICB), are most effective in select patient populations which express high PD-L1 or harbor high tumor mutation burdens and infiltrating immune cells [4, 5].

Adoptive cellular therapy (ACT) involves isolation and ex vivo expansion of cytotoxic immune cells, with or without genetic modification, for infusion into patients with cancer and may provide a new therapeutic option for patients who are not responsive to standard treatments [6]. Currently, there are several ACTs proposed for cancer therapy such as tumor-infiltrating lymphocytes (TILs), chimeric antigen receptor (CAR)- or T cell receptor (TCR)-modified T cells and cytotoxic innate lymphocytes such as cytokine-induced killer (CIK) cells, γδ-T or natural killer T cells [6–10]. Despite breakthrough success in targeting B cell leukemia and lymphoma [11], clinical success for ACT in solid tumors is limited due to challenges in obtaining sufficient numbers of tumor-reactive T cells and the immunosuppressive tumor microenvironment [12, 13]. The cell surface expressed programmed cell death 1 (PD-1) receptor has been implicated in tumor immune evasion of many cancer types through mediating inhibitory signals upon engagement of its ligand, PD-L1, expressed on tumors [14–17]. Indeed, the success of ICB antibodies targeting this pathway has led to clinical reduction of tumor size and improvements in overall patient survival, but as mentioned above, the response rates remain low [18–21].

Previously we have demonstrated that ex vivo expanded human peripheral blood T cells expressing CD3, without CD4, CD8, and NK T cell marker expression, termed double negative T (DNT) cells, have potent activity against lung cancer and leukemia cells in patient-derived xenograft (PDX) models [22–25]. We have developed protocols allowing for large-scale ex vivo expansion of clinical grade DNT cells and demonstrated that allogeneic DNT cells expanded from healthy donors are able to target a broad range of cancer cells in a donor non-restricted manner in vitro and in PDX models. Moreover, infusion of allogeneic DNT cells did not induce a host-versus-graft reaction nor cause graft versus host disease [24, 26]. These unique features of DNT cells make them different from conventional T cells and support their potential use as a new “off-the-shelf” ACT for cancers [26]. Based on these findings, a first-in-human clinical trial using ex vivo expanded DNT cells from healthy donors to treat high risk acute myeloid leukemia has been initiated (NCT03027102).

Whereas the anti-cancer activity of DNT cells has been demonstrated, little is known about the presence of DNT cells in patient lung tumors and how immune checkpoint blockade may regulate them. Here, we show that DNT cells are found amongst TILs of lung cancer patients and express PD-1. We further demonstrate that DNT cell therapy can inhibit the growth of late-stage established lung cancers in xenograft models and that addition of anti-PD-1 therapy further augments DNT cell-mediated anti-tumor function and increases their infiltration into tumor xenografts. Together, these data support the use of DNT cells as adoptive cellular therapy for NSCLC either alone or in combination with anti-PD-1 and show, for the first time, that anti-PD-1 antibody can increase tumor infiltration of adoptively transferred DNT cells.

## Methods

### Patient lung tissue sample collection and analysis

Resected human lung cancer tissue, cancer adjacent normal tissue (3–5 cm away from tumor) and grossly normal appearing lung tissue (> 10 cm away from tumor) from treatment naïve NSCLC patients were collected upon informed patient consent approved (NO.2016–408) by Ethics Committee, The First Hospital of Jilin University. Patient demographics are shown in Additional file 1: Table S1. Upon receiving samples, tissues were micro-dissected on ice and digested in a HBSS solution supplied with collagenase I and DNase I (Collagenase I: 100u/ml, DNase I: 1μg/ml) at 37 °C for 30 min. Dissected tissue suspensions were washed in cold PBS and filtered through cell strainers for single cell suspension. Red blood cell lysis was performed if necessary. T cell analysis of lung cancer tissue were performed using antibodies against human CD45 (clone HI30), CD3 (UCHT1), CD4 (SK3), CD8 (RPA-T8), PD-1 (EH12.1), CD45RA (HI100), CD27 (O323) and analyzed on BD LSR Fortessa and Canto Plus flow cytometer. Further details of all the antibodies used in this study are shown in Additional file 1: Table S2).

### T cell expansion

DNT cells were expanded as previously reported [22, 24]. Briefly, heparinized lung cancer patient or healthy donor derived peripheral blood was collected upon informed consent approved (#05–0221) by Research Ethics Board, University Health Network. CD4 and CD8 cells were depleted by using CD4- and CD8-antibody depletion cocktails (Stem cell Technologies) and DNT cell enriched population were cultured on anti-CD3 (OKT3, 5μg/ml) coated plates for 3 days in RPMI-1640 supplemented with 10% FBS and 250 unit /ml IL-2 (Proleukin, Novartis Pharmaceuticals). Cells were maintained in fresh media containing IL-2 (250 unit/ml), OKT3 (100 ng/ml) on day 7, 10, 12 and 14. Purity and PD-1 expression were analyzed by flow cytometry (Thermo Fisher Attune NxT, or BD Accuri C6) at indicated time points using anti-human antibodies CD3 (HIT3a), CD4 (RPA-T4), CD8 (SK1), PD-1 (EH12.2H7).

### Co-culture and cytotoxicity assay

Lung cancer cell lines NCI-H460, A549, and NCI-H520, were obtained from ATCC. Lung adenocarcinoma patient-derived xenograft cell line XDC137 was derived as previously reported [27, 28]. All the NSCLC cells were maintained in DMEM/F12(Gibco) supplemented with 10% FBS and screened for PD-L1 expression using anti-PD-L1 (29E.2A3) antibody by flow cytometry. A549 cell line were transduced with lentiviral PD-L1 (EX-OL03086-LX304) or GFP (EX-EGFP-LX304) expression vectors, respectively (both from GeneCopoeia). For DNT cell PD-1 induction assays, 1 × 10^5^ DNT cell were cultured alone or with 1 × 10^5^ NSCLC cells in 6-well plates for 1–5 days at 37 °C with 5% of CO2 in RPMI with 10% FBS and analyzed for PD-1 expression by flow cytometry. For intracellular cytokine staining in the absence of additional stimulation, DNT cells were incubated with Protein Transport Inhibitor Cocktail (eBiosciences) for 4 h prior to staining. For cytotoxicity assays, NCI-H460, XDC137, A549-control, A549-PD-L1 cell lines were labelled with 5 μM DiO (Life Technologies) dye and co-cultured with effector cells at different effector: target (E: T) ratios for 12-16 h. In some assays, anti-PD-1 (Nivolumab, 10μg/ml) or isotype control (human IgG4, 10μg/ml) were incubated for 30 min at room temperature with DNT cells prior to co-culture. Co-cultured cells were collected and lung cancer cytotoxicity was detected by TO-PRO-3 (life Technologies) dye incorporation and detected by flow cytometry by gating on DiO^+^ labelled cells. % specific killing by DNT cells was calculated by the formula: % Specific killing = $\frac{\%{DiO}^{+} TO- PRO-{3^{+}}_{with\  DNT}-\%{DiO}^{+} TO- PRO-{3^{+}}_{with out\  DNT}}{100-\%{DiO}^{+} TO- PRO-{3^{+}}_{with out\  DNT}}\times 100$ %.

### In vivo xenograft mice experiments

NOD.Cg-Prkdcscid Il2rgtm1Wjl/SzJ (NSG) mice were maintained at UHN animal facility. 6–8-week-old males were irradiated (250 cGy) 1 day prior to tumor inoculation. To establish late-stage xenograft models, 1 × 10^6^ NCI-H460 or XDC137 cells were injected subcutaneously in both flanks of NSG mice in a solution of 50% Matrigel. After the tumor volume reached ~100mm^3^, tumor bearing mice were treated 3 times with 2 × 10^7^ DNT, or CD8 T cells, or with PBS as controls through subcutaneous (s.c.) peritumoral injection or intravenous (i.v.) tail-vein injection, with or without 10 mg/kg anti-PD-1 (Nivolumab, *Bristol*-*Myers)*, intraperitoneal (i.p.) injections, starting one day before DNT cell transfer and repeated every 5 days to the end of the experiment. To facilitate DNT cell survival, all treatment groups were supplemented with human recombinant IL-2 (10^4^ U / dose, twice per week to the end of the experiment). Tumors were measured using digital calipers and tumor volumes were calculated using the formula length × width [2] × 0.5. When humane endpoint was reached according to institutional guidelines (tumor diameter of 1 .5cm), mice were sacrificed for humane reasons and tumor bearing mice survival curves were plotted.

### Histology and immunohistochemistry analysis

Tumor xenografts were fixed in 10% Formalin and paraffin embedded tissues were sent to the Applied Molecular Profiling Laboratory at Princess Margaret Cancer Centre for H&E or anti-human CD3 antibody staining. Sections were digitally scanned and analyzed using Aperio Image-scope (Leica Biosystems). Necrotic areas observed in the H&E stained tissues were quantified by determining percent of necrotic area per whole tumor area. Density of CD3^+^ infiltrating DNT cells were evaluated by positive CD3 staining pixel density per whole tumor area.

### Xenograft tumor infiltrating lymphocyte analysis

To analyze tumor infiltrating DNT cells, xenograft tumors were resected and digested as described above. Tumor tissues were filtered through 40-um nylon mesh cell strainers and red blood cells were lysed if needed. Necrotic debris were removed using a Dead Cell removal Kit (Miltenyi). Cells were stained with anti-human CD45 (HI30), NKG2D (1D11), DNAM-1 (11A8), PD-1 (EH12.2H7). For intracellular staining, single cell suspensions were stimulated with PMA/ionomycin cocktail followed by protein transport inhibition (eBiosciences) for 4 h and stained with IFN-γ (B27), TNF-α (Mab11), GranzymeB (GB11) and Perforin (B-D48). For CD107a analysis, anti-CD107a (H4A3) was added to intracellular stimulation cocktail. Frequency of infiltrating cells determined by total CD45^+^ cells in tumor x percentage of marker positive frequency.

### Statistical analysis

All graphs and statistical analysis were generated using GraphPad Prism 5. Unpaired student *t-*test was used when comparing two groups and 1-way ANOVA were used in comparing three groups. *, *p* < 0.05; **, *p* < 0.01; ***, *p* < 0.001 indicate significance between groups. Error bars represent mean ± SEM.

## Results

### DNT cells infiltrate patient lung adenocarcinoma and have cytotoxic function

**Fig. 1:**
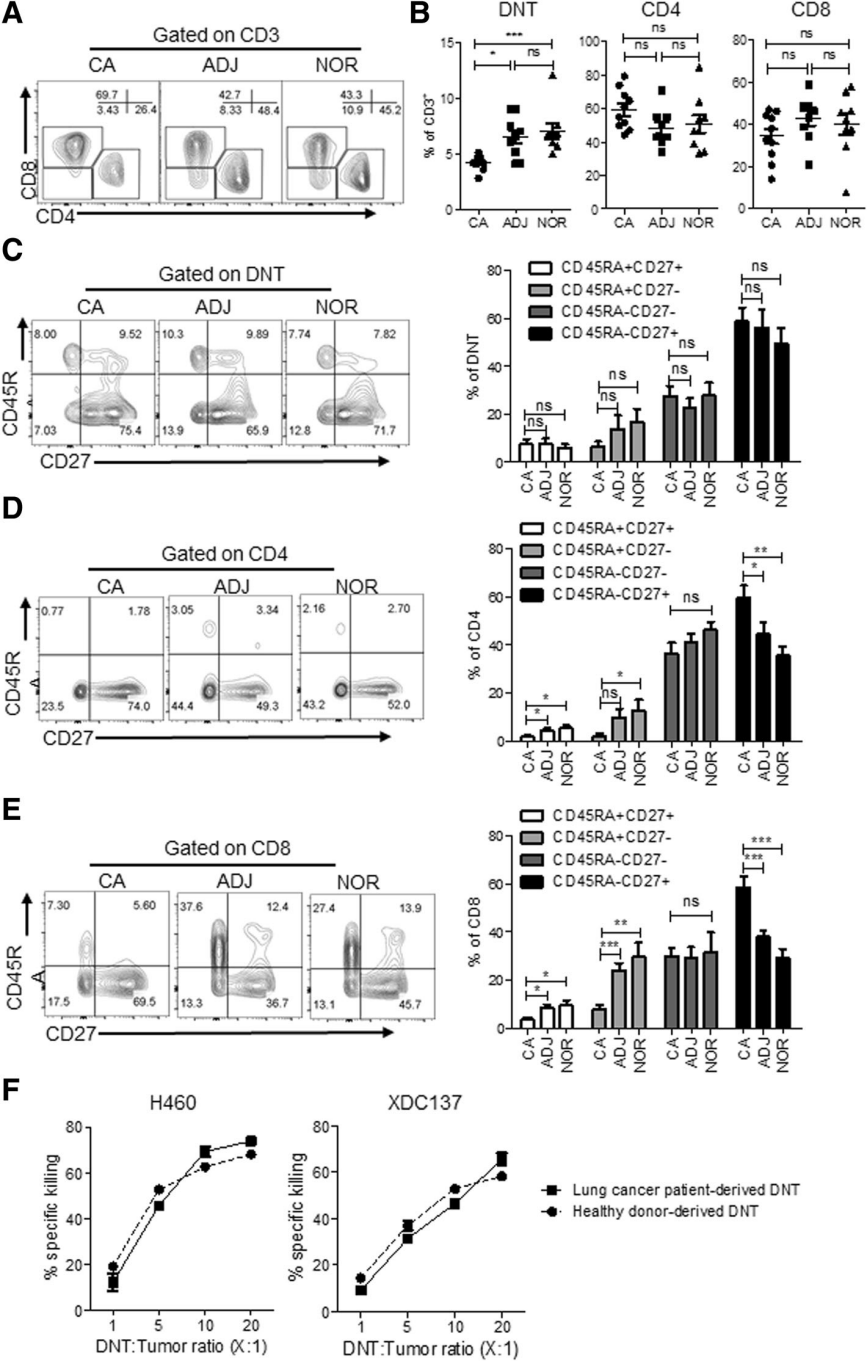
DNT cells infiltrate lung cancer and are cytotoxic to lung cancer cells. Flow cytometric analysis of T cells from different lung tissue compartments, cancer (CA), adjacent (ADJ), or grossly normal lung tissue (NOR), resected from lung cancer patients (*n* = 10). **a** Representative contour plots of lung tissue-derived T cell subsets by gating on CD3^+^ cells. **b** Frequency of lung tissue derived T cell subsets, each point represents data from an individual patient. *p < 0.05, **p < 0.01, ***p < 0.001 by one-way ANOVA. Representative flow cytometry plot and cumulative frequency of DNT (**c**), CD4 (**d**) and CD8 (**e**) T cell subsets expressing CD45RA and CD27, presented as mean ± SEM of 9 evaluable patients. *p < 0.05, **p < 0.01, ***p < 0.001 by two-tailed unpaired t-test relative to cancer (CA) tissue. **f** Lung cancer patient-derived or healthy donor-derived DNT cells were co-cultured with indicated lung cancer cell lines at various DNT cell to tumor ratios. % specific killing of target cells is shown. The results represent two independent experiments each with triplicate cultures

To explore the role of DNT cells in human lung cancer, we analyzed treatment naïve resected lung adenocarcinoma tissue (Additional file 1: Table S1). Single cell suspensions were generated from resected tumor tissue as well as from matching adjacent and grossly normal appearing tissue and analyzed for T cell presence. Flow cytometric analysis detected a population of DNT cells and conventional CD4^+^ and CD8^+^ T cells (Fig. 1a). Interestingly, whereas comparable levels of CD4^+^ and CD8^+^ T cells were observed in normal (NOR), adjacent (ADJ) or tumor tissue (CA), DNT cell frequency was significantly reduced in tumor tissues compared to adjacent or normal lung tissues (CA:4.2 ± 0.2% vs ADJ:6.5 ± 0.6% and NOR:7.0 ± 0.7%, respectively; Fig. 1b). Based on co-staining of CD45RA and CD27, human T cells can be broadly categorized as effector memory (CD45RA^−^CD27^−^) or central memory (CD45RA^−^CD27^+^) subsets [29–31]. We found that tumor infiltrating DNT cells were predominantly central memory cells with no significant differences observed between different tissues (Fig. 1c). However, we did observe a significantly higher frequency of central memory phenotype amongst CD4 and CD8 T cells within cancer tissue relative to adjacent and normal lung tissue (Fig. 1d and e).

Given the presence of DNT cells in tumor tissue, we determined whether DNT cells derived from lung cancer patients have anti-tumor function. Using our well-established DNT cell expansion protocol, by which we have previously expanded DNT cells from peripheral blood of both leukemia patients [22] and healthy donors [24], we attempted to selectively expand DNT cells from tumor samples, but failed, possibly due to the low frequency of DNT cells obtained from tumors and/or exhaustion of the obtained DNT cells. However, DNT cells expanded from peripheral blood of lung cancer patients resulted in high purity (> 90%) but with a lower yield than those derived from healthy donors (Additional file 2: Figure S1). Importantly, DNT cells expanded from both lung cancer patients and healthy donors displayed potent and comparable cytotoxicity against established lung cancer cell line, NCI-H460, and patient xenograft-derived cell line XDC137 (Fig. 1f). Next, we compared the potency of anti-tumor activity mediated by CD4, CD8 and DN T cells expanded from the same donor in in vitro killing assays against the two cell lines and found that while all expanded T cell subsets showed cytotoxicity towards lung cancer cell lines, DNT cells induced the highest degree of cytotoxicity (Additional file 2: Figure S2A).

### Ex vivo expanded DNT cells from healthy donors can target advanced late-stage lung cancer xenografts

**Fig. 2:**
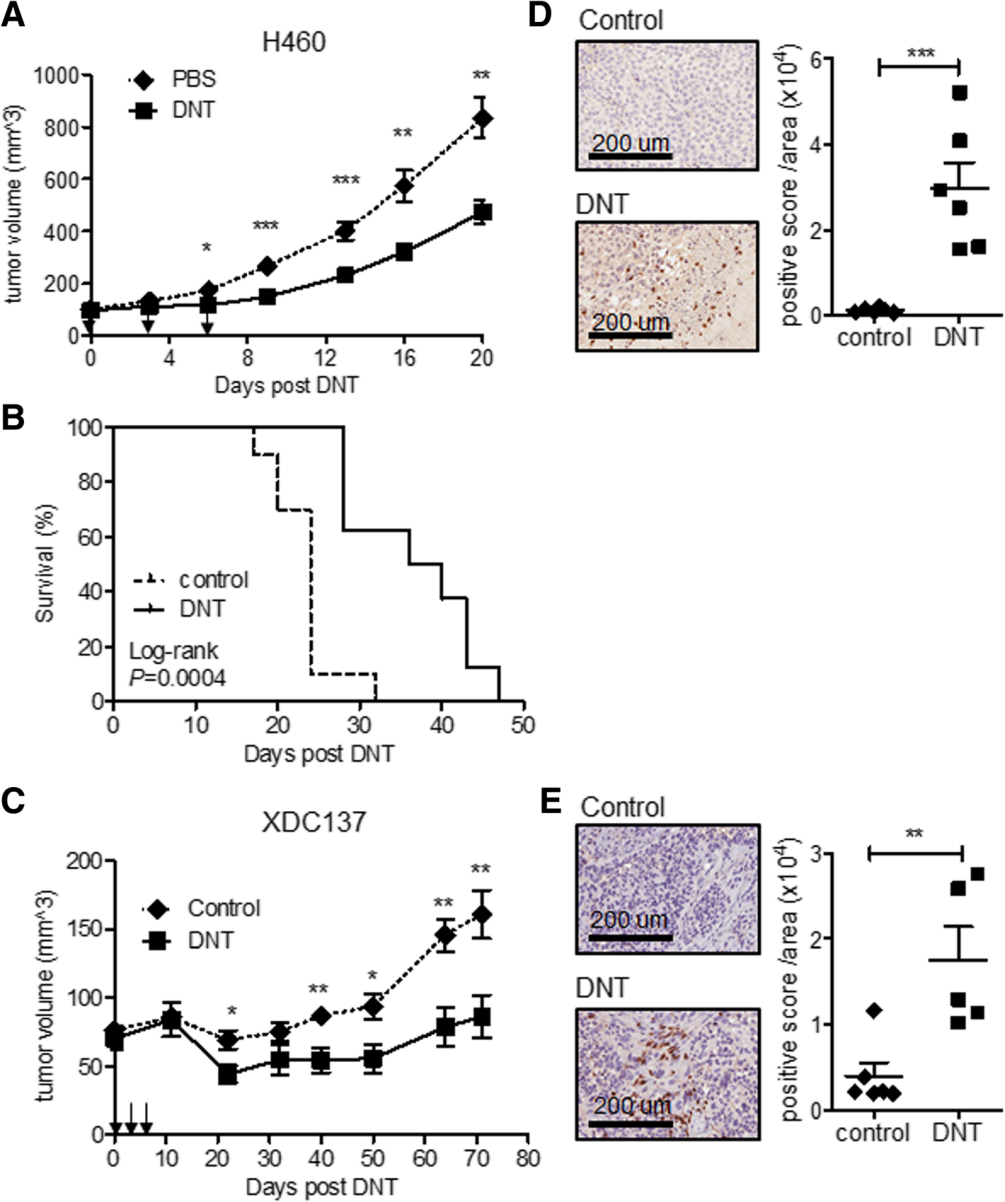
Ex vivo expanded DNT cells inhibit late stage tumor growth in xenograft models. NSG mice were inoculated subcutaneously with NCI-H460 (**a, b, d**) or XDC137 (**c** and **e**) in 50% Matrigel solution and grown to ~100mm^3^. After tumors were established, tumor bearing mice were randomized into groups and treated with peritumoral injection of IL-2 with or without DNT cells on day 0, 3 and 6. (**a** and **c**) Tumor volume was measured at indicated time points. Arrows indicate DNT cell treatments. Results represent one of three independent experiments, each consisting of 5 mice per treatment group (**a**), or one experiment consisting of 3 mice per treatment group (**c**). **b** Survival of mice receiving IL-2 (control) or IL-2 + DNT cells (DNT) **d** and **e.** Immunohistochemistry staining with anti-human CD3 antibody on resected tumor xenografts. Representative sections of CD3^+^ DNT cells within tumor xenograft from both groups are shown at 21 days for NCI-H460 xenografts (**d**) and at 71 days for XDC137 xenografts (**e**) Quantified CD3^+^ staining density of whole xenograft sections, as determined by digital analysis of positive stain per area analyzed. Each dot represents one mouse and horizontal bars represent the mean ± SEM. Data shown are representative of 2 separate experiments. *p < 0.05, **p < 0.01, ***p < 0.001, by two-tailed unpaired t-test (**a, c, d** and **e**) or log-rank (**b**)

To determine whether DNT cells can target late-stage lung cancer in vivo, we generated two late-stage xenograft models. An NSCLC established cell line NCI-H460 and a patient-derived adenocarcinoma xenograft cell line XDC137 were inoculated subcutaneously (s.c.) into the flanks of sublethally irradiated NSG mice and let grown to ~100mm^3^. Tumor-bearing mice were then treated subcutaneously with 3 peritumoral injections of ex vivo expanded DNT cells or CD8 T cells in 3–4 days intervals. For the more aggressive NCI-H460 model, the PBS treated control tumor reached end-point by 20 days post treatment (Fig. 2a). However, DNT cell treatment resulted in a significant reduction of tumor growth as early as 6 days post 1st DNT cell injection. At 20 days post DNT cell treatment NCI-H460 tumor volume was reduced by 43.3 ± 15.9%, from 834.2 ± 234.8 mm^3^ in the control group to 473.2 ± 132.9 mm^3^ in the DNT cell treated group (Fig. 2a). In contrast, injection of an equal number of CD8 T cells was not able to reduce tumor growth during this observation period (Additional file 2: Figure S2B). Additionally, DNT cell-mediated inhibition of tumor growth led to a significant increase in the survival of NCI-H460 tumor-bearing mice, with a humane endpoint extending from median 24 days to 38 days (Fig. 2b). Though patient-derived xenograft model XDC137 grew much slower than NCI-H460, with humane endpoint not being reached by 71 days of observation, DNT cell treatment significantly reduced XDC137 xenograft volume from 160.8 ± 39.5mm^3^ in the PBS control group to 86.2 ± 34.8mm^3^ in the DNT cell treated group (Fig. 2c), resulting in a 46.4 ± 21.6% reduction in tumor volume. These results show that adoptive transfer of healthy donor-derived DNT cells can significantly inhibit the growth of both aggressive and slow growing lung cancer xenografts. As DNT cells were found in lung cancer patient TILs, we next determined whether DNT cells would be detectable within tumor xenografts at experimental endpoints. Using immunohistochemical staining for human CD3^+^ cells, we detected DNT cells infiltrating both aggressive xenograft, NCI-H460 (Fig. 2d) and slower growing xenograft, XDC137 (Fig. 2e), at days 21 and day 71, respectively.

### Tumor infiltrating and ex vivo expanded DNT cells express PD-1

**Fig. 3:**
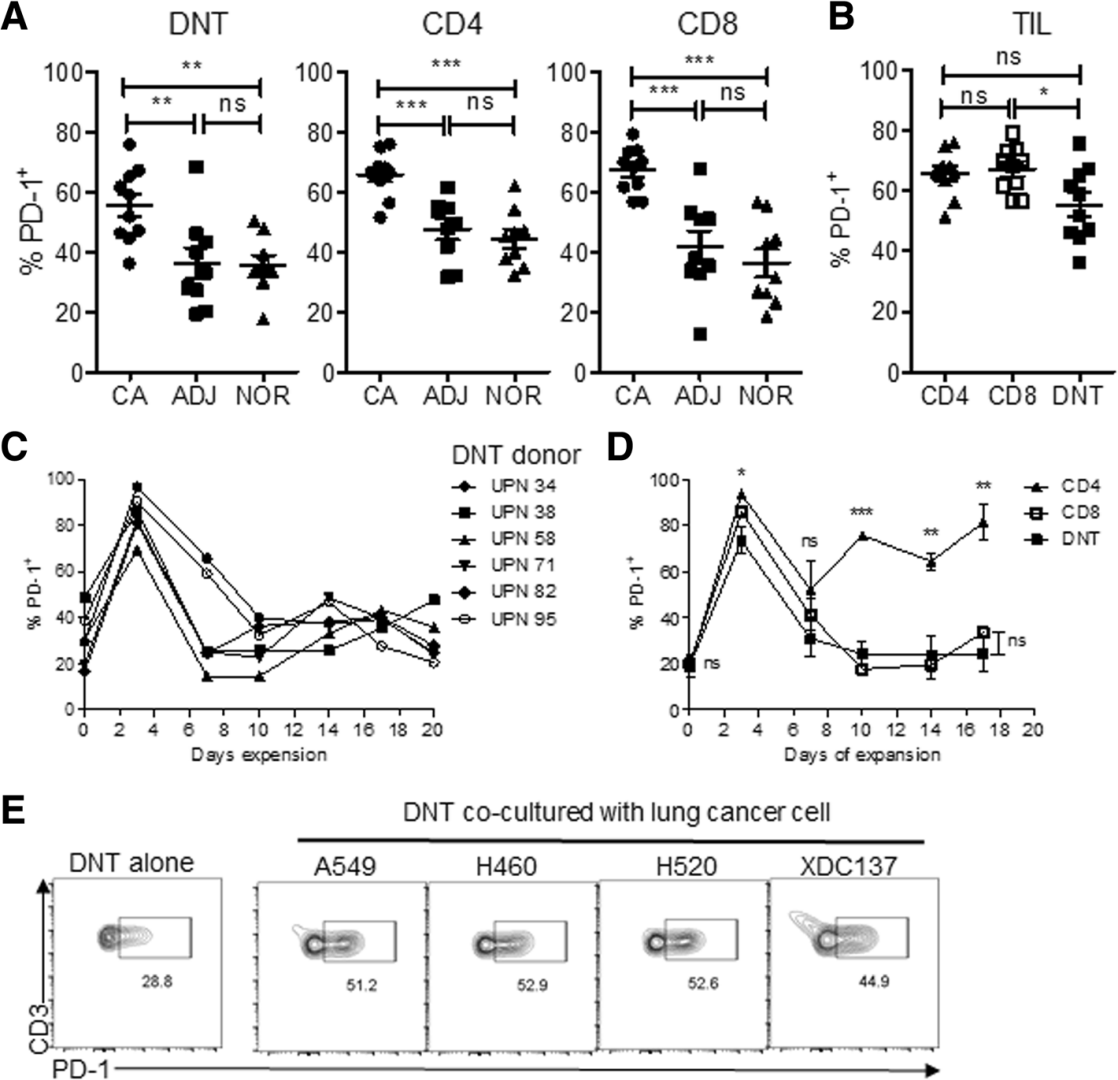
DNT cells upregulate PD-1 during interaction with NSCLC. Flow cytometric analysis of PD-1 expression on T cells from resected tissue compartment of lung cancer patients’ cancer (CA), adjacent (ADJ), or normal lung tissue (NOR) (n = 10). **a** Frequencies of PD-1^+^ T cell subsets in patient lung tissue. **b** Comparison of tumor infiltrating PD-1^+^ T cells subsets in cancer tissue. Each symbol represents an individual patient, bars represent mean value. *p < 0.05, **p < 0.01, ***p < 0.001 by one-way ANOVA. **C.** Time course of PD-1 expression on expanded DNT cells, results of 6 independent experiments done with DNT cells expanded from 6 different donors. **d** Time course of PD-1 expression on expanded CD4, CD8 and DNT cells, results shown as mean ± SEM expanded from 3 different donors. *p < 0.05, **p < 0.01, ***p < 0.001, by two-tailed unpaired t-test. **e** PD-1 expression on DNT cells cultured alone or with various NSCLC cell lines for 48 h. One representative result of two independent experiments is shown

With the observation that significantly fewer DNT cells were found in the patient TILs than in adjacent or normal tissue (Fig. 1b), we hypothesized that the immunosuppressive tumor microenvironment may prevent DNT cell infiltration. Consistent with this hypothesis, PD-1 was expressed on DNT cells within resected lung tissue, similar to that seen for CD4^+^ and CD8^+^ T cells (Fig. 3a). Further, a significantly higher proportion of DNT cells expressed PD-1 within tumors compared to adjacent or normal tissue (CA: 55.5 ± 11.7% vs ADJ: 36.1 ± 14.5% and NOR: 35.5 ± 9.1%). Though tumor-infiltrating DNT cells expressed PD-1, they were the least frequent PD-1^+^ T cell subset and showed the most variability in PD-1 expression compared to CD4^+^ and CD8^+^ T cells (CD4: 65.8 ± 7.1%, CD8: 67.2 ± 7.2%, DNT: 55.5 ± 11.7%, Fig. 3b).

Since patient-derived DNT cells induced a similar level of cytotoxicity against lung cancer cells as those from healthy donors (Fig. 1f), and DNT cells expanded from healthy donors possess features allowing them to be used as an “off-the-shelf” ACT [32], we utilized healthy donor DNT cells to understand the role of PD-1 expression on DNT cells. Prior to expansion, PD-1 expression varied amongst donors (Fig. 3c, day 0 of expansion). Upon expansion, donor DNT cells followed a similar expression profile: sharply increasing expression of PD-1 at day 3 of culture then gradually returning to baselines by day 17 (Fig. 3c). We observed a similar trend of PD-1 expression for CD8 T cells expanded in this manner. In contrast, CD4 T cells maintained a significantly higher level of PD-1 expression than DNT and CD8 T cells from day 10 until the end of the expansion culture (Fig. 3d). Given that PD-1 expression was higher in tumor infiltrating DNT cells than those in adjacent or normal lung tissues (Fig. 3a), and lung cancer cell lines express different levels of PD-L1 (Additional file 2: Figure S3A), we determined if co-culture of DNT cells with lung cancer cells was sufficient to induce PD-1 expression. Consistent with the observations in patients, in vitro coculture with 4 different PD-L1^+^ lung cancer cell lines (A549, H460, H520 and XDC137, Additional file 2: Figure S3A), all resulted in a significant increase in PD-1^+^ DNT cells when compared with DNT cells cultured alone (Fig. 3e and Additional file 2: Figure S3B). PD-1 induction was not dependent on the level of PD-L1 expression on lung cancer cells as H520 expressed the lowest level of PD-L1 (Additional file 2: Figure S3A) but induced a similar level of PD1^+^DNT cells as H460 which showed a very high level of PD-L1 expression (Fig. 3e and Additional file 2: Figure S3A). Prolonged co-cultures with lung cancer cells did not further increase PD-1^+^ DNT cells for any given cell line (Additional file 2: Figure S3B). Co-culture with lung cancer cell lines also increased intracellular expression of IFNγ and TNFα in DNT cells (Additional file 2: Figure S4), suggesting the activation of these T cells by lung cancer cells.

### Anti-PD-1 treatment enhances DNT cell-mediated anti-tumor activity

**Fig. 4:**
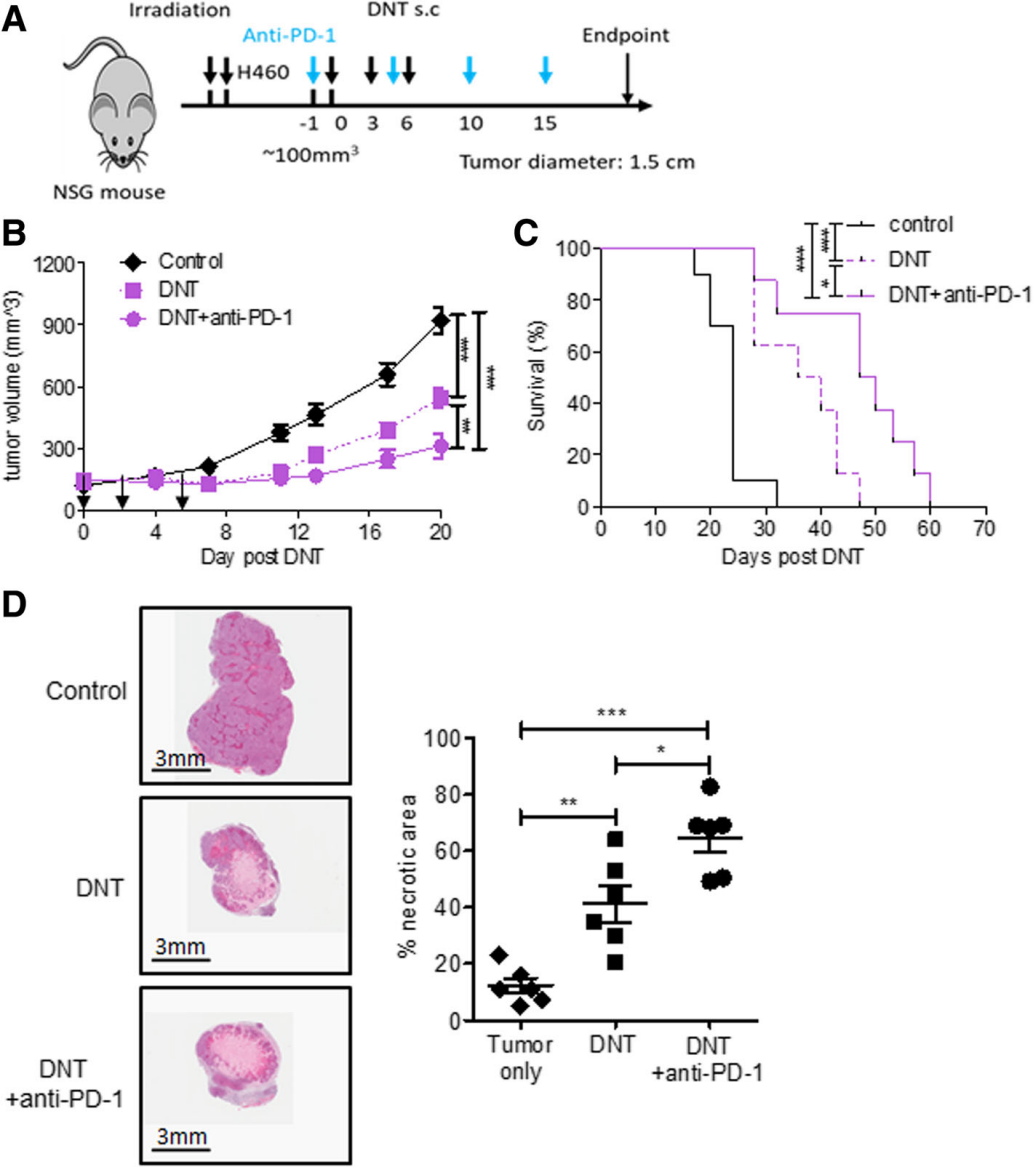
Anti-PD-1 antibody enhances the efficacy of DNT cell-mediated inhibition of late-stage tumor growth. NSG mice were inoculated subcutaneously with NCI-H460 in 50% Matrigel solution and grown to ~100mm^3^. After tumors were established, tumor bearing mice were randomized and received peritumoral injection of DNT cells and IL-2 on day 0, 3 and 6, without or with anti-PD-1 antibody (10 mg/kg repeated every 5 days i.p., starting one day prior to 1st DNT cell infusion). **a** Schematic diagram of the treatment protocol of NCI-H460 xenograft model. **b** Tumor volume was measured at indicated time points (*n* = 8 for each group). **c** Survival of the mice treated with PBS control or DNT cells with or without anti-PD-1 (n = 8 for each group). **d**. Representative **h**&**e** staining of xenografts from indicated treatment groups 9-days post DNT cell infusion (*n* = 6 for each group; 3 tumors each with 2 random sections). **e** Percent necrotic area in tumors from indicated treatment groups calculated by histological analysis (n = 6 for each group; 3 tumors each with 2 random sections). Representative results shown as mean and SEM of 2 separate experiments. *p < 0.05, **p < 0.01, ***p < 0.001, by two-tailed unpaired *t* test (**b**), log-rank test (**c**)**,** or one-way ANOVA (**e**)

With the propensity of DNT to upregulate PD-1 and cytokines expression in the presence of lung cancer, we sought to determine if addition of anti-PD-1 may augment DNT cell-mediated anti-tumor activity in vivo. To observe whether anti-PD-1 can benefit adoptive DNT therapy in vivo, PD-L1 expressing NCI-H460 lung cancer cell line was subcutaneously implanted and established to ~ 100 mm^3^ and DNT cells, with or without anti-PD-1, were administered using two methods, either locally by s.c. peritumoral injection or systemically by intravenous (i.v.) tail vein injection as shown schematically in Fig. 4a and Additional file 2: Figure S5A, respectively. Anti-PD-1 treatment alone had no effect on tumor growth compared with PBS treated controls (Additional file 2: Figure S6) and consistent with Fig. 2a, peritumoral infusion of DNT cells significantly reduced NCI-H460 tumor volume from 922.1 ± 164.2 mm^3^ in the control group to 546.5 ± 125.7 mm^3^ in the DNT cell treated group, resulting in a 40.7 ± 13.6% reduction in tumor volume. Interestingly, the combination of DNT cell injection with anti-PD-1 resulted in an additional 43.1 ± 29.4% reduction of tumor volume (from 546.5 ± 125.7 mm^3^ in the DNT cell alone treated group to 310.7 ± 160.9 mm^3^ in the combination group) by day 20 (Fig. 4b). Similarly, systemic i.v. infusion of DNT cells also significantly reduced NCI-H460 tumor volume from 1017.49 ± 246.2 mm3 in the control group to 572.5 ± 186.5 mm3 in the DNT cell treated group, resulting in a 43.7 ± 18.3% reduction in tumor volume, and the combination therapy of i.v. inoculated DNT cells and anti-PD-1 treatment resulted in an additional 32.6 ± 20.0% reduction in tumor volume (from 572.5 ± 186.5 mm^3^ in the DNT cell alone treated group to 385.9 ± 114.3 mm^3^ when in combination) by day 20 (Additional file 2: Figure S5B). Importantly, combination therapy prolonged the survival of both s.c. peritumoral inoculated DNT cell treated mice from median 38 days to 48.5 days (Fig. 4c) and i.v. inoculated DNT cell treated mice from median 33 days to 38 days (Additional file 2: Figure S5C). Analysis of hematoxylin and eosin (H&E) stained tumor tissue shortly after DNT treatment revealed that though tumor size remained similar (181.0 ± 53.7 mm^3^ for DNT cell treated vs 152.2 ± 54.7 mm^3^ for DNT cell and anti-PD-1 treated), anti-PD-1 significantly increased the proportion of necrotic area detected within tumors from mice receiving combination treatment (64.9 ± 11.7% vs 41.3 ± 14.5%; Fig. 4d), with a similar result observed for i.v. inoculated DNT cells (42.1 ± 10.4% vs 22.4 ± 7.2%; Additional file 2: Figure S5D). These results suggest that DNT cells inhibit tumor growth by actively targeting tumor cells and causing tumor necrosis, and that this activity was enhanced by anti-PD1 therapy. Overall, these results show that addition of anti-PD-1 augments the ability of DNT cells to reduce tumor growth and increase survival of mice.

### Anti-PD-1 treatment increases DNT cell infiltration into tumor xenografts

**Fig. 5:**
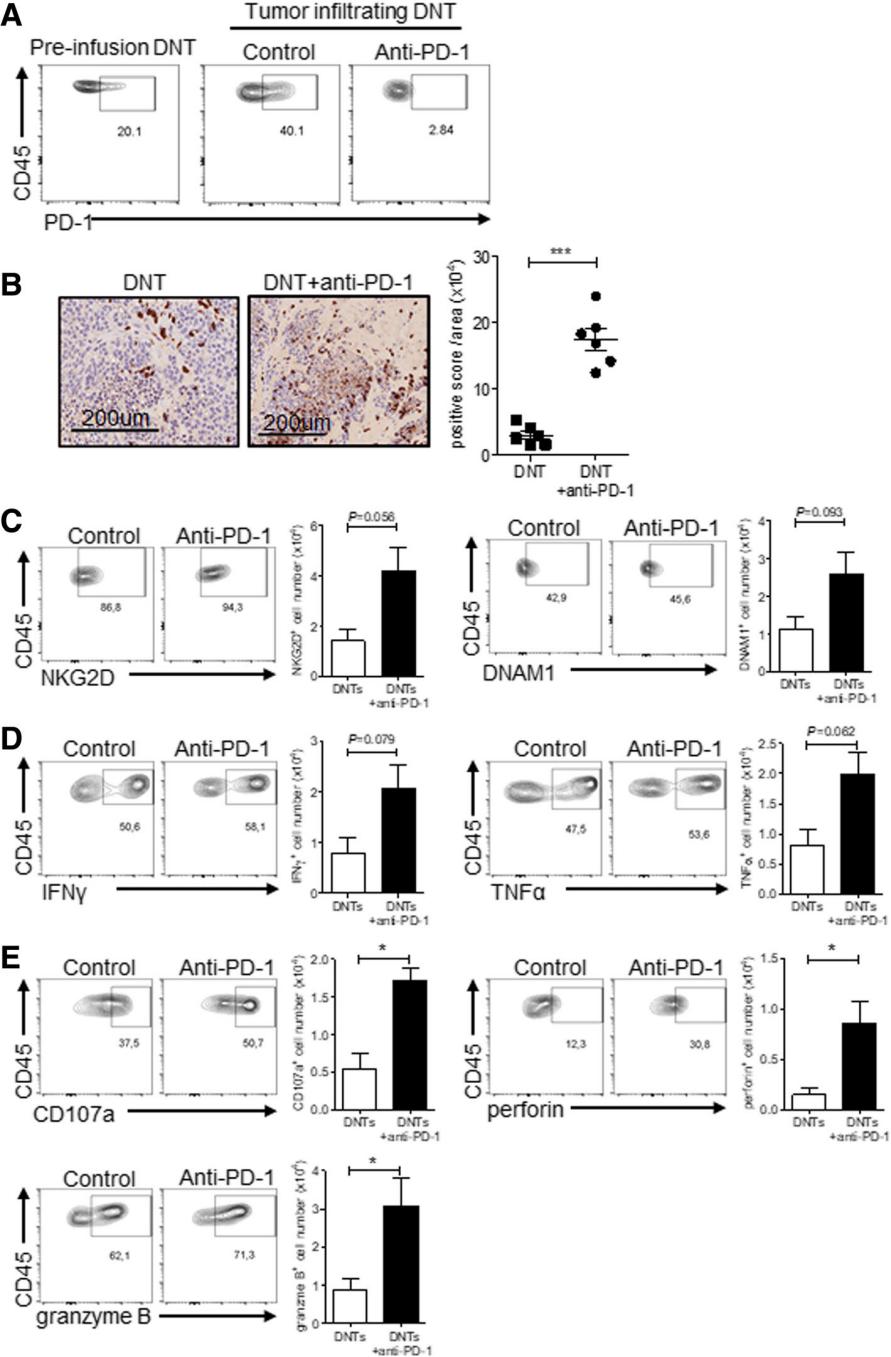
Anti-PD-1 antibody enhances infiltration of cytotoxic DNT cells into tumor xenografts. Tumor-bearing NSG mice received peritumoral injection of DNT cells with or without anti-PD1 treatment. **A.** Representative flow cytometric analysis of DNT cells pre-infusion and tumor infiltrating DNT cells 21 days post infusion. The data shown represent results from 2 independent experiments. **b** Immunohistochemistry analysis of DNT cells. Nine days post DNT cell infusion, tumor xenografts were harvested and stained with anti-human CD3 antibody and quantified by Aperio Image-scope. Representative staining and analysis of tumor infiltrating DNT cells in indicated treatment groups are shown. Each dot represents one mouse and horizontal bars represent the mean ± SEM. Data shown are representative of 2 separate experiments. **c-e** Flow cytometry analysis of tumor infiltrating DNT cells. Frequency of NKG2D^+^ or DNAM-1^+^ DNT cells (**c**)**.** IFNγ^+^ and TNFα^+^ DNT cells (**d**), perforin^+^, granzyme B^+^ and CD107a^+^ DNT cells (**e**). Representative results shown as mean ± SEM from 3 tumors of 2 separate experiments are shown. (*p < 0.05 by two-tailed unpaired *t*-test)

To understand how anti-PD-1 augmented DNT cell-mediated tumor growth inhibition, we first determined whether the presence of anti-PD-1 altered in vitro cytotoxicity of DNT cells to lung cancer cell lines expressing different levels of PD-L1 (Additional file 2: Figure S7A). We found that addition of anti-PD-1 to the cocultures did not alter DNT cell cytotoxicity towards lung cancer cell lines H460, XDC137 and A549 natively expressing PD-L1, but significantly increased killing of PD-L1 overexpressing cell line A549-PD-L1 (Additional file 2: Figure S7B). To analyze how anti-PD-1 enhanced DNT cell treatment towards lung cancer xenografts in vivo we analyzed tumor infiltrating DNT cells post treatment. Consistent with PD-1 induction on DNT cells by lung cancer in vitro (Fig. 3e), flow cytometric analysis of xenograft infiltrating DNT cells showed a 2-fold increase in PD-1 expression compared to DNT cells prior to infusion (Fig. 5a). Further, anti-PD-1 treatment abrogated PD-1 expression on xenograft infiltrating DNT cells as shown by the lack of staining using anti-PD1 clone EH12.2H7 that recognizes a Nivolumab shared epitope of PD-1 [33, 34] (Fig. 5a), suggesting that the Nivolumab treatment effectively blocked the PD-1 epitope on tumor infiltrating DNT cells.

To determine whether anti-PD-1 treatment affects tumor infiltration of DNT cells, we quantified DNT cell infiltration of tumor xenografts by histological analysis. Mice receiving combination treatment of DNT cells and anti-PD-1 antibody had a 5.9 ± 1.2-fold increase in the number of tumor infiltrating DNT cells relative to mice that received DNT cells alone (Fig. 5b). Similarly, i.v. infusion of DNT cells also resulted in a 1.7 ± 0.3-fold increase in DNT cells accumulating in tumor xenografts (Additional file 2: Figure S5E). These data indicate that anti-PD-1 treatment can increase the accumulation of DNT cells in tumor tissue. We next analyzed whether anti-PD-1 treatment could alter the phenotype of tumor infiltrating DNT cells. To this end, tumor infiltrating DNT cells were isolated from mice receiving different treatments and expression of cytolytic molecules known to be involved in DNT cell anti-tumor responses were analyzed by flow cytometry [24, 25, 35]. We found that DNT cells expressing NKG2D and DNAM1 were present in both control and anti-PD-1 treated mice but were more abundant in mice receiving combination therapy than those receiving DNT cells alone, though differences did not reach statistical significance (Fig. 5c). Similarly, mice that received anti-PD-1 showed a greater number of TNFα^+^ and IFNγ^+^ DNT cells in the tumor (Fig. 5d). Importantly, consistent with the cytotoxic nature of DNT cells, anti-PD-1 treatment significantly increased the frequency of CD107a^+^, perforin^+^, and granzyme B^+^ DNT cells within tumors (Fig. 5e). These data suggest that anti-PD-1 treatment increases the accumulation of DNT cells within tumors expressing molecules involved in anti-tumor responses.

## Discussion

Adoptive cellular therapy based on DNT cells emerges as a promising therapeutic option for hematological and lung malignancies [22–26]. Here we show that adoptive transfer of DNT cells significantly inhibited growth of late-stage lung tumor xenografts and enhanced the survival of recipient mice. Moreover, we show that anti-PD-1 increased the accumulation of cytotoxic DNT cells within tumor xenografts. These results collectively demonstrate the potential of DNT cells to benefit NSCLC patients, particularly those receiving ICB treatment with limited response due to lack of TILs.

Tumor infiltrating CD8^+^ and CD4^+^ T cells remain an important predictor of patient outcomes and responsiveness to anti-PD-1 therapy, with recent discoveries highlighting a role for TCF7^+^CD8^+^ T cells in predicting responsiveness [36–39]. However, the role of non-conventional T cells, such as DNT cells, in solid tumor remains largely unexplored. By examining lung cancer patients’ resected lung tissues we found that DNT cells were present within early stage lung adenocarcinoma (Fig.1 a and b) and exhibited a predominate central/effector memory phenotype (Fig. 1d). Further, whereas we observed no significant difference between conventional CD4 and CD8 T cells in their infiltration, a lower frequency of DNT cells was found infiltrating tumors relative to adjacent and normal tissue, suggesting that the tumor microenvironment may be more hostile to DNT cells (Fig. 1b). Of note, whereas significantly higher frequencies of central memory CD4^+^ and CD8^+^ T cells were found in cancer tissue relative to adjacent and normal lung tissue (Fig. 1d and e), this was not the case for DNT cells (Fig. 1c). While our attempts to directly measure tumor infiltrating DNT cell cytotoxicity against lung cancer failed due to the limited number of DNT cells available for expansion, indirect evidence from DNT cells expanded amongst total TILs from pancreatic and glioma patient tumors showed intracellular IFNγ and TNFα expression upon stimulation by autologous tumor [40, 41], suggesting that tumor infiltrating DNT cells are likely to be cytotoxic.

We have previously shown that allogeneic DNT cells do not induce host-versus-graft rejection nor cause graft-versus-host disease [24, 26]. Consistent with the non-allogeneic nature of DNT cells, peripheral blood DNT cells derived from lung cancer patients exhibited similar cytotoxicity to that of healthy donor derived DNT cells against the same lung cancer cells (Fig. 1f). Additionally, a report showed that lung cancer patients have fewer circulating DNT cells in peripheral blood than healthy donors [42] and that fewer DNT cells were expanded from lung cancer patients (Additional file 2: Figure S1B). Furthermore, we found that expanded DNT cells exerted a greater cytotoxicity against lung cancer in vitro compared to CD4 and CD8 T cells from the same donor (Additional file 2: Figure S2A). Together, these findings suggest that the use of healthy donor DNT cells is more practical and will make DNT-cell therapy more readily available.

Importantly, DNT cells, but not CD8 T cells, significantly inhibited late-stage H460 lung tumor growth in vivo (Fig. 2a and Additional file 2: Figure S2B) and prolonged survival of tumor bearing mice (Fig. 2b and d). In the case of slow growing patient-derived xenograft cell line, XDC137, DNT cell treatment limited the growth of the tumor for over 70 days of the observation period and DNT cells were found infiltrating the tumor at this time point (Fig. 2e), suggesting that adoptive transfer of DNT cells could lead to a long-lasting anti-tumor immunity. Interestingly, though adoptive cellular therapy shows promise in clinical trials, rarely do preclinical studies show complete tumor regression in xenograft models [43–46]. Similarly, DNT cell therapy significantly inhibited tumor growth but did not eradicate late-stage lung cancer xenografts. This may be due to the lack of other components of the immune system in immunodeficient mice which may not support memory T cell formation or may be due to the immunosuppressive tumor microenvironment [47].

Given the role of the tumor microenvironment in regulating T cells [12, 13], we found that tumor-infiltrating DNT cells had a higher expression of PD-1 relative to adjacent and normal tissue (Fig. 3a). Consistent with this observation, DNT cells co-cultured with lung cancer cells increased PD-1 expression (Fig. 3e and Additional file 2: Figure S3B). Additionally, xenograft infiltrating DNT cells also showed higher PD-1 expression compared to pre-infusion cells (Fig. 5a). Collectively, our findings are consistent with the observation that tumor recognition and activation of T cells lead to upregulation of PD-1 [17, 48] and suggest that expression of PD-1 on DNT cells is regulated in a similar manner. Interestingly, patient-derived tumor infiltrating DNT cells expressed a lower level of PD-1 than conventional CD4^+^ and CD8^+^ T cells (Fig. 3b). In line with this, we found that stimulation of CD4 T cells in vitro resulted in sustained PD-1 expression, which differs from what was observed for DNT and CD8 T cells (Fig. 3d). These findings show differences in PD-1 regulation between T cell subsets and suggest the possibility that DNT cells may be more resistant to tumor microenvironmental changes in vivo than conventional T cells.

Observations from patients responsive to ICB suggest that blocking PD-1 greatly increased the number and function of CD8^+^ T cells infiltrating the tumor bed [49]. Interestingly, we found that the addition of anti-PD-1 to DNT and lung cancer cell co-cultures only increased killing of PD-L1 over expressing cells but not the lung cell lines natively expressing PD-L1 (Additional file 2: Figure S7). Though initially surprising, this observation was consistent with results published by others using gamma/delta T cells and CIK cells [44, 46] and suggests that tumor natively expressed PD-L1 may not have enough density to alter innate T cell function in vitro. Similar to observations in patients receiving ICB, we found that anti-PD-1 blockade also led to greater numbers of DNT cells within tumors (Fig. 5b and Additional file 2: Figure S5E), suggesting that DNT cells were regulated by the PD-1/PD-L1 pathway. Whether anti-PD-1 blockade increased the ability of DNT cells to migrate to xenografts or survive within xenografts was not directly explored, but given the role of PD-1 engagement in regulating T cell activation [14] and apoptosis [15, 16], and that DNT cells could infiltrate tumors in the absence of ICB (Fig. 2d and e), anti-PD-1 blockade may allow for continued DNT cell survival within tumors.

Tumor recognition by DNT cells was shown to be dependent on ligation of NKG2D and DNAM1 receptors by innate ligands preferentially expressed on malignant cells [24, 25]. In addition to increasing the number of DNT cells within tumor xenografts, we found that anti-PD-1 treatment resulted in increased NKG2D^+^ and DNAM1^+^ DNT cells, capable of cytolytic granule secretion (Fig. 5). This increase in tumor-recognizing DNT cells within xenografts coincided with an increase in tumor necrosis (Fig. 4d and Additional file 2: Figure S5D), supporting direct engagement and lysis of lung cancer xenografts by DNT cells. Indeed, addition of anti-PD-1 to adoptively transferred DNT cells significantly enhanced DNT cell-mediated tumor inhibition and prolonged the survival of tumor bearing mice (Fig. 4 and Additional file 2: Figure S5). Taken together, these data support the notion that combination therapy of anti-PD-1 and DNT cells is beneficial to DNT cell therapy of solid tumors such as lung cancer.

Our results show that ex vivo expanded DNT cells can infiltrate and inhibit the growth of late-stage lung cancer in xenograft models. Given the similarity between DNT cells derived from lung cancer patients and healthy donors, the non-allogeneic “off-the-shelf” nature of DNT cells may be ideal for adoptive cell therapy in lung cancer. This contrasts other adoptive cellular therapy combination strategies that utilize autologous CIK^44^, which are difficult to grow from patients, or antigen specific T cells [43] which may be prone to resistance by tumor antigen loss [5]. Given the innate recognition mechanisms utilized by DNT cells, which do not rely on traditional peptide-HLA recognition [24], DNT cell therapy is less likely affected by the known primary or acquired resistances to ICB such as a low tumor mutation burden, lack of tumor reactive T cells [4, 5] or loss of HLA [50]. Further, as DNT cells show benefit from addition of ICB, DNT cell therapy can be used as an adjunct to patients already receiving immune checkpoint blockade and may be ideal for patients characterized as having “immune deserts”.

## Conclusions

We demonstrated for the first time that patient-derived tumor infiltrating lymphocytes contained a lower frequency of DNT cells with a higher expression of PD-1 relative to normal lung tissue. Our data show that DNT cells are cytotoxic to lung cancer cells in vitro and can inhibit the progression of late-stage lung cancer in vivo. DNT cell treatment in combination with anti-PD-1 resulted in increased DNT cell-mediated anti-tumor activity in vivo by increasing the frequency of effector DNT cells in tumors. These results highlight the effect of DNT cells and combinatorial potential of DNT cellular therapy with anti-PD-1 checkpoint blockade for the treatment of lung cancer.

## Additional files

Additional file 1: **Table S1.** Patients’ clinical characteristics. Ten newly diagnosed lung cancer patients who were treated with surgery alone were enrolled in this study. The patient age, gender, pathological classification and stage are shown. **Table S2.** List of flow cytometric antibodies used in the study. (DOCX 15 kb)

Additional file 2: **Figure S1.** Purity and yield of lung cancer patient- and healthy donorderived DNT cells. Lung cancer patient-derived DNT cells (*n* = 4) or healthy donor-derived DNT cells (*n* = 5) were expanded as described in the Materials and Methods and analyzed for their phenotype (A) and yield (B) 14 days after expansion. **Figure S2.** A. Healthy donor derived DNT, CD4 and CD8 T cells were co-cultured with indicated lung cancer cell lines at various T cell to tumor cell ratios for 12-14 h. % specific killing of target cells is shown. The results represent 3 independent experiments from 3 different donors, each with triplicate cultures. Results shown as mean ± SEM. *p < 0.05, **p < 0.01, ***p < 0.001 indicate the differences in the cytotoxicity of DNT versus CD8 T cells at indicated E:T ratios by two-tailed unpaired t-test. B. NSG mice were inoculated subcutaneously with NCI-H460 in 50% Matrigel solution and grown to ~ 100 mm3. After tumors were established, tumor bearing mice were randomized into groups and treated with peritumoral injection of IL-2 with or without DNT or CD8 T cells from the same donor on day 0, 3 and 6. Tumor volume was measured at indicated time points. Results represent one of two independent experiments, each had 5 mice per treatment group. Results shown as mean ± SEM. *p < 0.05, **p < 0.01 show the difference in tumor volume at indicated timepoints between DNT and CD8 treated groups by two-tailed unpaired student t-test. **Figure S3.** A. The expression of PD-L1 on NSCLC cell lines. NSCLC cell lines were stained with either anti-human PD-L1 (blue histograms) or control (red histograms), numbers represent MFI. B. Time course of PD-1 expression on expanded DNT cells, cultured alone or with various NSCLC cell lines for varying time points. Results represent the data obtained from 2 different donors. **Figure S4.** Intracellular cytokine analysis of DNT cells, either cultured alone or with various NSCLC cell lines. Expanded DNT cells were culture alone or co-cultured with indicated NSCLC cell lines for 48 h and prior to analysis of indicated intracellular cytokines. % INF-γ and TNF-α positive DNT cells are shown. **Figure S5.** Anti-PD-1 antibody enhances efficacy of DNT cell mediated antitumor response and DNT cell tumor infiltration in late stage tumor xenograft model. NSG mice were inoculated subcutaneously with NCI-H460 in 50% Matrigel solution and tumors were allowed to grow to ~ 100 mm3. After tumors were established, tumor bearing mice were randomized and received intravenous injection of IL-2 (control treatment) or DNT cell plus IL-2 on days 0, 3 and 6. Additionally, some mice received PBS or anti-PD-1 antibody (10 mg/kg repeated every 5 days i.p., starting one day prior to 1st DNT cell infusion to the end of the experiment). A. Schematic diagram of the treatment protocol of NCI-H460 xenograft model. B. Tumor volume were measured at indicated time points (*n* = 10 for each group). C. Humane end point survival of treated mice (n = 10 for each group). D. Representative H&E staining of xenografts from indicated treatment groups 9 days post DNT cell infusion and percent necrotic area in tumors from indicated treatment groups calculated by histological analysis. E. Immunohistochemical analysis of CD3+ human T cells in tumor xenografts 9 days post DNT cell infusion. Representative staining and analysis of tumor infiltrating DNT cells in indicated treatment groups. Results shown as mean ± SEM, from untreated, DNT (*n* = 6) or DNT plus anti-PD1 treatment groups (n = 6). Results shown are representative of 2 separate experiments. **p < 0.01, ***p < 0.001, by two-tailed unpaired t-test (B), by log-rank test (C) or by one-way ANOVA (D and E). **Figure S6.** Treatment with anti-PD-1 alone has no effect on NCI-H460 xenograft growth or mouse survival. A. NSG mice were inoculated subcutaneously with NCI-H460 in 50% Matrigel solution and grown to ~ 100 mm3, and treated with 10 mg/kg anti-PD-1 or PBS i.p. every 5 days to the end of the experiment. Tumor volume and recipient survival were monitored (*n* = 5 for each group). **Figure S7.** A. The expression of PDL1 on NSCLC cell line H460, XDC137, A549 control vector and A549 PD-L1 overexpressing cell line. NSCLC cell lines were stained with either anti human PD-L1(blue histograms) or control (red histograms), values represent MFI. B. DNT cells were co-cultured with indicated lung cancer cell lines at a 5 to 1 DNT cell to tumor cell ratio. % specific killing of target cells is shown. Results shown as mean ± SEM. The results represent 3 independent experiments each with triplicate cultures. **p < 0.01, by two-tailed unpaired t-test. (PDF 827 kb)

## Abbreviations

ACT: Adoptive cellular therapy
ADJ: Adjacent tumor tissue
CA: Cancer tissue
CAR: Chimeric antigen receptor
CIK: Cytokine-induced killer
DNAM 1: DNAX accessory molecule 1
DNT: Double negative T cell
E:T: Effector to target
FBS: Fetal bovine serum
HBSS: Hanks’ balanced salt solution
i.v.: Intravenous
ICB: Immune checkpoint blockade
IFNγ: Interferon gamma
NKG2D: Natural-killer group 2
NOR: Grossly normal appearing tissue
NSCLC: Non-small cell lung cancer
PBS: Phosphate-buffered saline
PD-1: Programmed cell death protein 1
PD-L1: Programmed cell death-ligand 1
PDX: Patient-derived xenograft
s.c.: Subcutaneously
TCR: T cell receptor
TILs: Tumor-infiltrating lymphocytes
TNFα: Tumor necrosis factor alpha
