# Supplementary material for: Targeting late-stage non-small cell lung cancer with a combination of DNT cellular therapy and PD-1 checkpoint blockade
Source: J Exp Clin Cancer Res. 2019 Mar 11;38:123. doi: 10.1186/s13046-019-1126-y (PMC6413451; doi:10.1186/s13046-019-1126-y)
Supplement: Supplementary file 1 — Table S1. Patients’ clinical characteristics. Ten newly diagnosed lung cancer patients who were treated with surgery alone were enrolled in this study. The patient age, gender, pathological classification and stage are shown. Table S2. List of flow cytometric antibodies used in the study. (DOCX 15 kb) [file 13046_2019_1126_MOESM1_ESM.docx]

**Supplementary Table S1.** **Patients’ clinical characteristics.** Ten newly diagnosed lung cancer patients who were treated with surgery alone were enrolled in this study. The patient age, gender, pathological classification and stage are shown.

| **Patient number** | **Gender** | **Age** | **Pathological classification** | **Pathological stage** |
| --- | --- | --- | --- | --- |
| 1 | Male | 46 | Adenocarcinoma | I B |
| 2 | Female | 59 | Adenocarcinoma | I A |
| 3 | Female | 54 | Adenocarcinoma | I A |
| 4 | Female | 57 | Adenocarcinoma | I A |
| 5 | Male | 62 | Adenocarcinoma | I B |
| 6 | Female | 62 | Adenocarcinoma | III A |
| 7 | Female | 63 | Adenocarcinoma | I A |
| 8 | Male | 62 | Adenocarcinoma | I B |
| 9 | Male | 45 | Adenocarcinoma | I B |
| 10 | Female | 60 | Adenocarcinoma | I A |

**Supplementary Table S2. List of flow cytometric antibodies used in the study.**

| Reagent | Source | Identifier |
| --- | --- | --- |
| CD45-PERCP-CY5.5 (clone: HI30) | BD Biosciences | Cat: 564105 |
| CD3-PE (clone: UCHT1) | BD Biosciences | Cat: 555333 |
| CD4-PECY7 (clone: SK3) | BD Biosciences | Cat: 557852 |
| CD8-V450 (clone: RPA-T8) | BD Biosciences | Cat: 560347 |
| PD1-BV605 (clone: EH12.1) | BD Biosciences | Cat: 563245 |
| Live/dead Fixable aqua-BV510 | Invitrogen | Cat: L34957 |
| CD45RA-APC-cy7 (clone: HI100) | Biolegend | Cat: 304128 |
| CD27-BV421 (clone: O323) | Biolegend | Cat: 302824 |
| CD3-PEcy7 (clone: HIT3a) | Biolegend | Cat: 300316 |
| CD4-FITC (clone: RPA-T4) | Biolegend | Cat: 300519 |
| CD8-APC (clone: SK1) | Biolegend | Cat: 344722 |
| PD1-PE (clone: EH12.2H7) | Biolegend | Cat: 329906 |
| CD45-FITC (clone: HI30) | Biolegend | Cat: 304006 |
| NKG2D-PE (clone: 1D11) | Biolegend | Cat: 320806 |
| DNAM1-APC (clone: 11A8) | Biolegend | Cat: 338312 |
| Perforin-PE (clone: B-D48) | Biolegend | Cat: 353304 |
| Granzyme B-AF647 (clone: GB11) | Biolegend | Cat: 515406 |
| IFN-y-PE (clone: B27) | Biolegend | Cat: 506507 |
| TNF-a-APC (clone: MAb11) | Biolegend | Cat: 502912 |
| CD107a-APC (clone: H4A3) | Biolegend | Cat: 328620 |
