# Supplementary material for: Targeting late-stage non-small cell lung cancer with a combination of DNT cellular therapy and PD-1 checkpoint blockade
Source: J Exp Clin Cancer Res. 2019 Mar 11;38:123. doi: 10.1186/s13046-019-1126-y (PMC6413451; doi:10.1186/s13046-019-1126-y)
Supplement: Supplementary file 2 — Figure S1. Purity and yield of lung cancer patient- and healthy donorderived DNT cells. Lung cancer patient-derived DNT cells (n = 4) or healthy donor-derived DNT cells (n = 5) were expanded as described in the Materials and Methods and analyzed for their phenotype (A) and yield (B) 14 days after expansion. Figure S2. A. Healthy donor derived DNT, CD4 and CD8 T cells were co-cultured with indicated lung cancer cell lines at various T cell to tumor cell ratios for 12-14 h. % specific killing of target cells is shown. The results represent 3 independent experiments from 3 different donors, each with triplicate cultures. Results shown as mean ± SEM. *p < 0.05, **p < 0.01, ***p < 0.001 indicate the differences in the cytotoxicity of DNT versus CD8 T cells at indicated E:T ratios by two-tailed unpaired t-test. B. NSG mice were inoculated subcutaneously with NCI-H460 in 50% Matrigel solution and grown to ~ 100 mm3. After tumors were established, tumor bearing mice were randomized into groups and treated with peritumoral injection of IL-2 with or without DNT or CD8 T cells from the same donor on day 0, 3 and 6. Tumor volume was measured at indicated time points. Results represent one of two independent experiments, each had 5 mice per treatment group. Results shown as mean ± SEM. *p < 0.05, **p < 0.01 show the difference in tumor volume at indicated timepoints between DNT and CD8 treated groups by two-tailed unpaired student t-test. Figure S3. A. The expression of PD-L1 on NSCLC cell lines. NSCLC cell lines were stained with either anti-human PD-L1 (blue histograms) or control (red histograms), numbers represent MFI. B. Time course of PD-1 expression on expanded DNT cells, cultured alone or with various NSCLC cell lines for varying time points. Results represent the data obtained from 2 different donors. Figure S4. Intracellular cytokine analysis of DNT cells, either cultured alone or with various NSCLC cell lines. Expanded DNT cells were culture alone or co-cultured with [file 13046_2019_1126_MOESM2_ESM.pdf]

Figure S1.

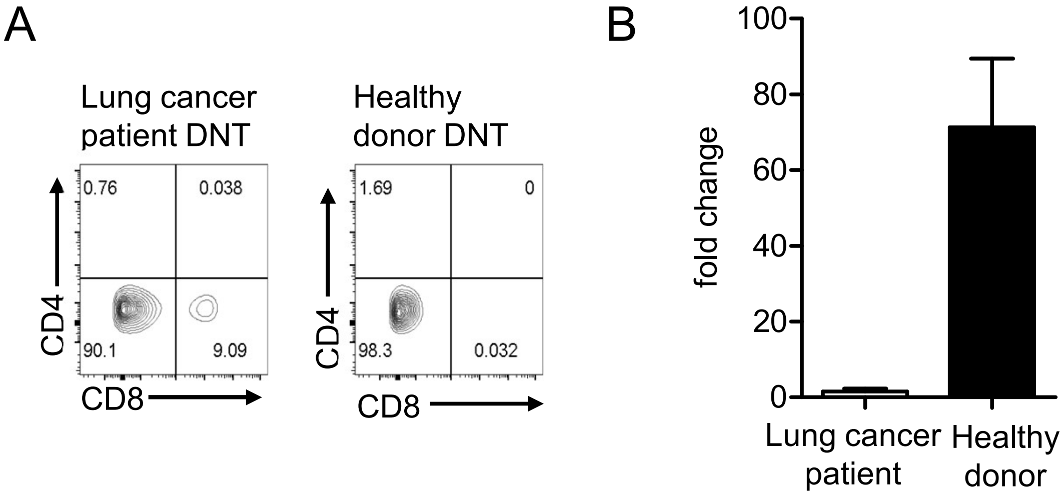

Figure S1. Purity and yield of lung cancer patient- and healthy donor-derived DNT cells. Lung cancer patient-derived DNT cells (n=4) or healthy donor-derived DNT cells (n=5) were expanded as described in the Materials and Methods and analyzed for their phenotype **(A)** and yield **(B)** 14 days after expansion.

Figure S2.

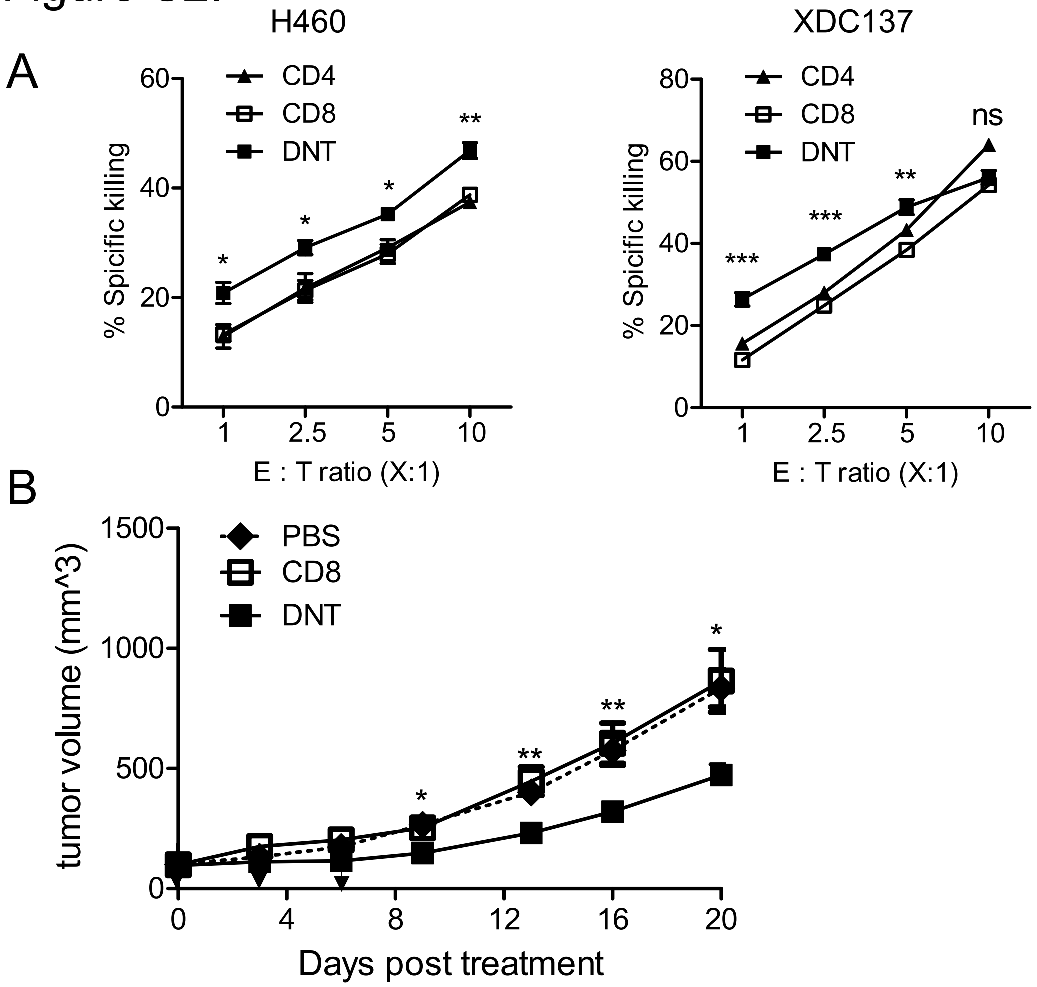

Figure S2. A. Healthy donor derived DNT, CD4 and CD8 T cells were co-cultured with indicated lung cancer cell lines at various T cell to tumor cell ratios for 12-14hr. % specific killing of target cells is shown. The results represent 3 independent experiments from 3 different donors, each with triplicate cultures. Results shown as mean ± SEM. *p<0.05, **p<0.01, ***p<0.001 indicate the differences in the cytotoxicity of DNT versus CD8 T cells at indicated E:T ratios by two-tailed unpaired t-test. **B**. NSG mice were inoculated subcutaneously with NCI-H460 in 50% Matrigel solution and grown to ~100mm3. After tumors were established, tumor bearing mice were randomized into groups and treated with peritumoral injection of IL-2 with or without DNT or CD8 T cells from the same donor on day 0, 3 and 6. Tumor volume was measured at indicated time points. Results represent one of two independent experiments, each had 5 mice per treatment group. Results shown as mean ± SEM. *p<0.05, **p<0.01 show the difference in tumor volume at indicated time-points between DNT and CD8 treated groups by two-tailed unpaired student t-test.

Figure S3.

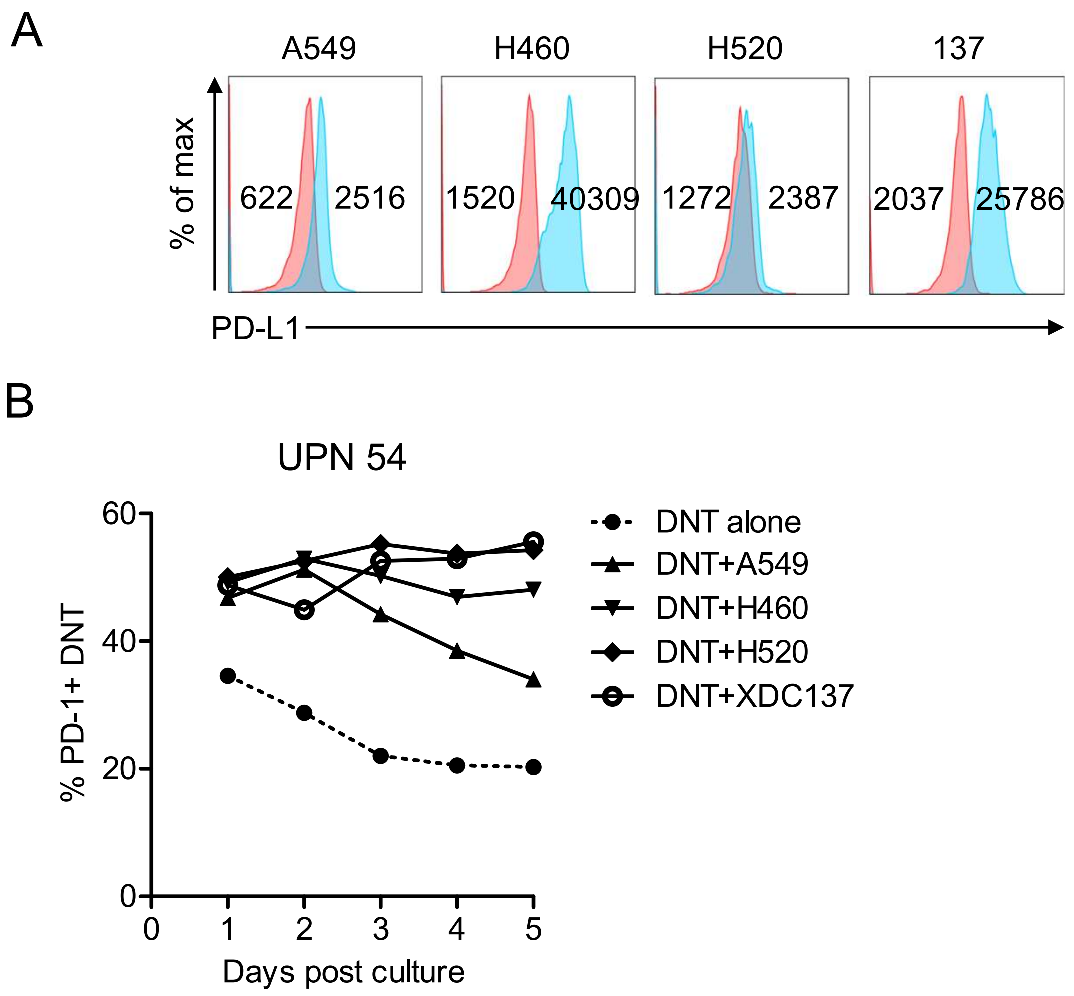

Figure S3. A. The expression of PD-L1 on NSCLC cell lines. NSCLC cell lines were stained with either anti-human PD-L1 (blue histograms) or control (red histograms), numbers represent MFI. **B.** Time course of PD-1 expression on expanded DNT cells, cultured alone or with various NSCLC cell lines for varying time points. Results represent the data obtained from 2 different donors.

Figure S4.

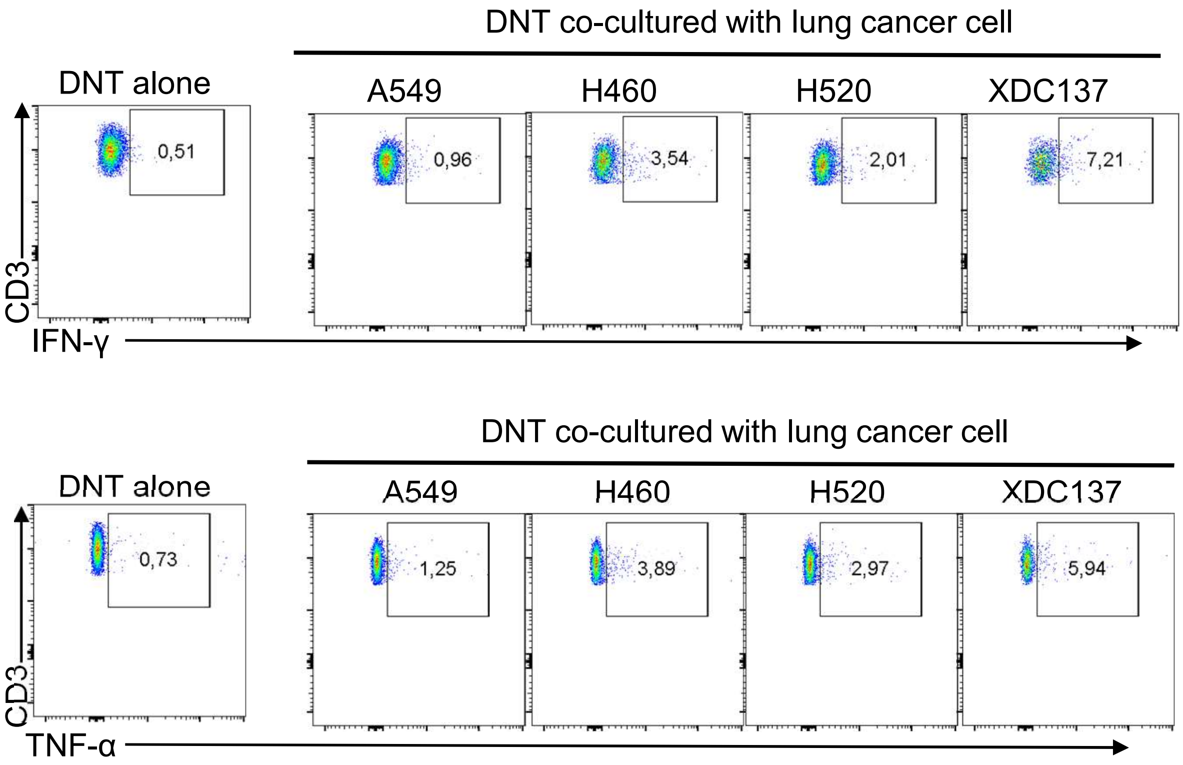

Figure S4. Intracellular cytokine analysis of DNT cells, either cultured alone or with various NSCLC cell lines. Expanded DNT cells were culture alone or co-cultured with indicated NSCLC cell lines for 48hr and prior to analysis of indicated intracellular cytokines. % INF-γ and TNF-α positive DNT cells are shown.

Figure S5.

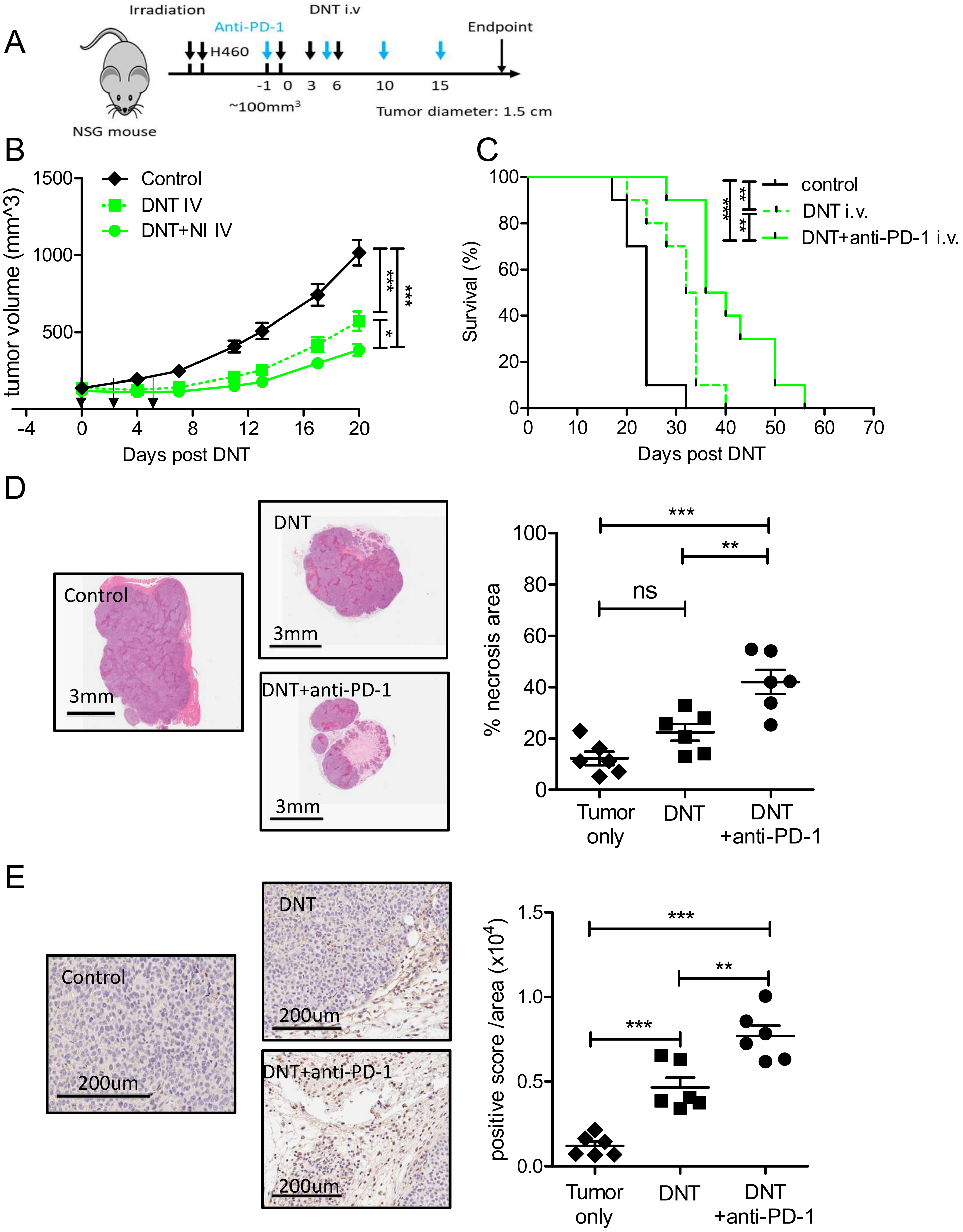
A
Irradiation
Anti-PD-1
DNT i.v
Endpoint
H460
-1 0 3 6 10 15
~100mm³
Tumor diameter: 1.5 cm
NSG mouse
B
tumor volume (mm^3)
Days post DNT
Control
DNT IV
DNT+NI IV
C
Survival (%)
Days post DNT
control
DNT i.v.
DNT+anti-PD-1 i.v.
D
Control
DNT
DNT+anti-PD-1
3mm
% necrosis area
ns
Tumor only
DNT
DNT +anti-PD-1
E
Control
DNT
DNT+anti-PD-1
200um
positive score /area (x10⁴)
Tumor only
DNT
DNT +anti-PD-1

Figure S5. Anti-PD-1 antibody enhances efficacy of DNT cell mediated anti-tumor response and DNT cell tumor infiltration in late stage tumor xenograft model. NSG mice were inoculated subcutaneously with NCI-H460 in 50% Matrigel solution and tumors were allowed to grow to ~100mm^3. After tumors were established, tumor bearing mice were randomized and received intravenous injection of IL-2 (control treatment) or DNT cell plus IL-2 on days 0, 3 and 6. Additionally, some mice received PBS or anti-PD-1 antibody (10mg/kg repeated every 5 days i.p., starting one day prior to 1st DNT cell infusion to the end of the experiment). **A.** Schematic diagram of the treatment protocol of NCI-H460 xenograft model. **B.** Tumor volume were measured at indicated time points (n=10 for each group). **C.** Humane end point survival of treated mice (n=10 for each group). **D**. Representative H&E staining of xenografts from indicated treatment groups 9 days post DNT cell infusion and percent necrotic area in tumors from indicated treatment groups calculated by histological analysis. **E.** Immunohistochemical analysis of CD3$^+$ human T cells in tumor xenografts 9 days post DNT cell infusion. Representative staining and analysis of tumor infiltrating DNT cells in indicated treatment groups. Results shown as mean ± SEM, from untreated, DNT (n=6) or DNT plus anti-PD1 treatment groups (n=6). Results shown are representative of 2 separate experiments. **p<0.01, ***p<0.001, by two-tailed unpaired t-test (**B**), by log-rank test **(C)** or by one-way ANOVA **(D and E).**

Figure S6.

A

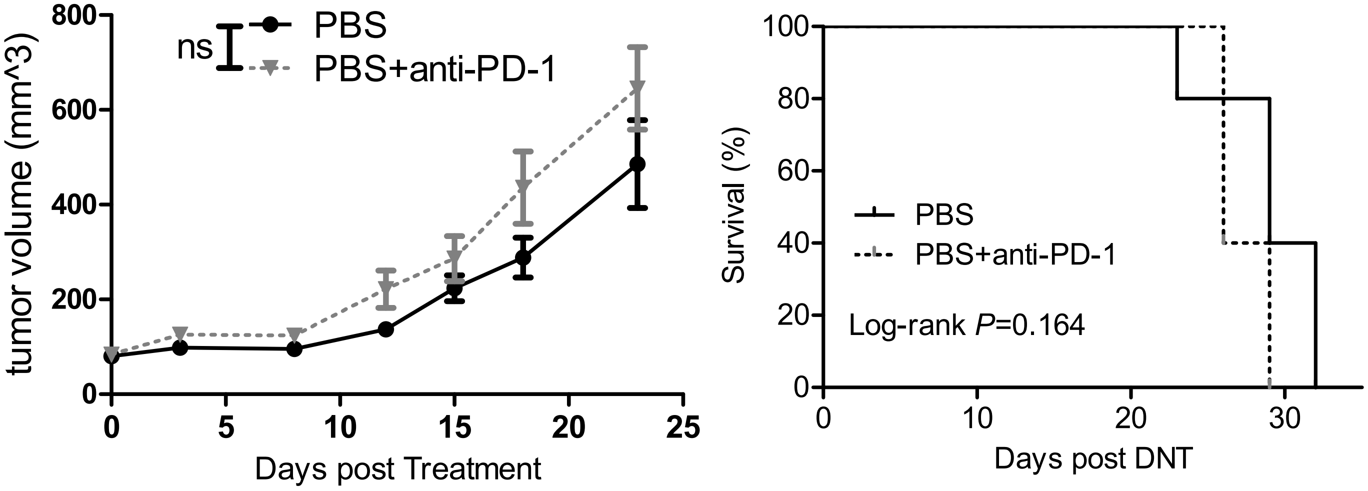

Figure S6. **Treatment with anti-PD-1 alone has no effect on NCI-H460 xenograft growth or mouse survival. A.** NSG mice were inoculated subcutaneously with NCI-H460 in 50% Matrigel solution and grown to ~100mm^3, and treated with 10mg/kg anti-PD-1 or PBS i.p. every 5 days to the end of the experiment. Tumor volume and recipient survival were monitored (n=5 for each group).

Figure S7.

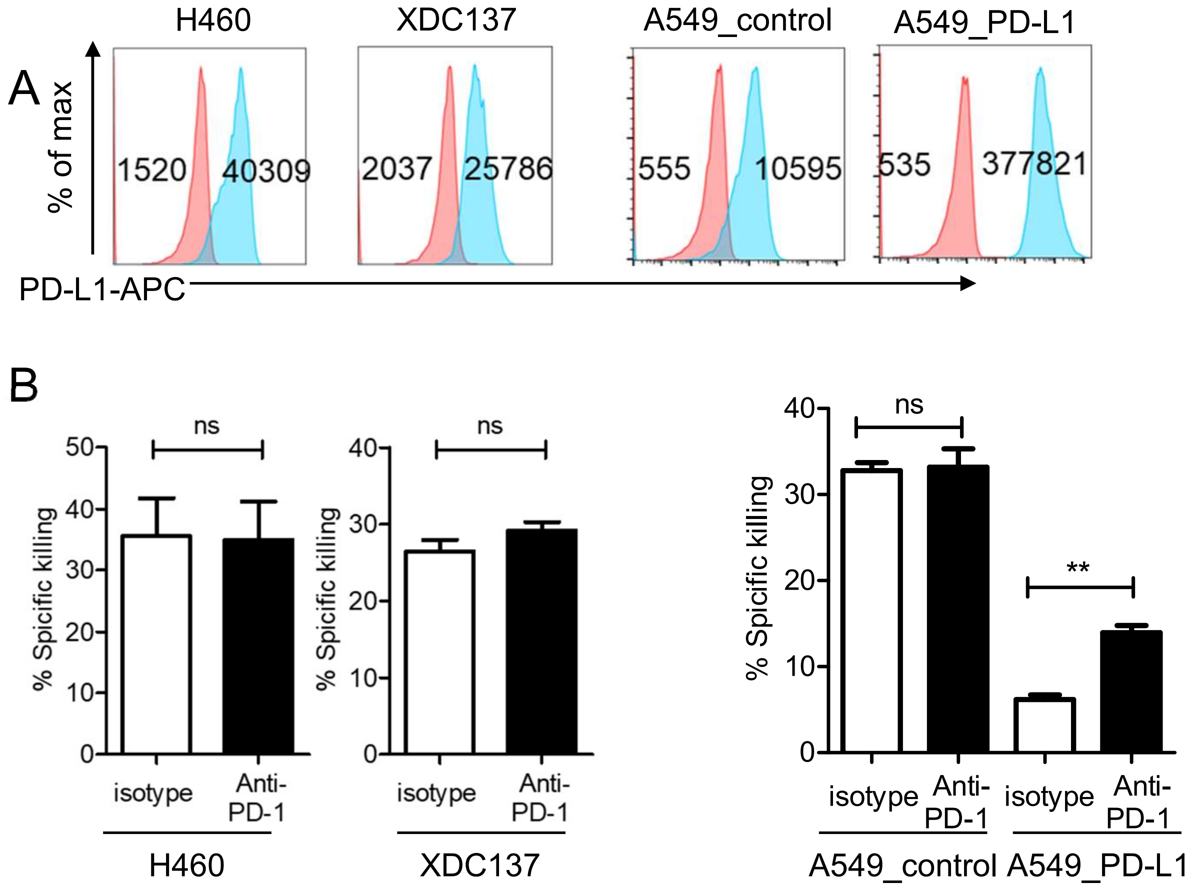

Figure S7. A. The expression of PDL1 on NSCLC cell line H460, XDC137, A549 control vector and A549 PD-L1 overexpressing cell line. NSCLC cell lines were stained with either anti human PD-L1(blue histograms) or control (red histograms), values represent MFI. **B.** DNT cells were co-cultured with indicated lung cancer cell lines at a 5 to 1 DNT cell to tumor cell ratio. % specific killing of target cells is shown. Results shown as mean ± SEM. The results represent 3 independent experiments each with triplicate cultures. **p<0.01, by two-tailed unpaired t-test.
